# Characterization of AQPs in Mouse, Rat, and Human Colon and Their Selective Regulation by Bile Acids

**DOI:** 10.3389/fnut.2016.00046

**Published:** 2016-10-10

**Authors:** Jonathan Yde, Stephen Keely, Qi Wu, Johan F. Borg, Natalia Lajczak, Aoife O’Dwyer, Peter Dalsgaard, Robert A. Fenton, Hanne B. Moeller

**Affiliations:** ^1^Department of Biomedicine, InterPrET Center, Aarhus University, Aarhus, Denmark; ^2^RCSI Education and Research Centre, Royal College of Surgeons in Ireland, Beaumont Hospital, Dublin, Ireland; ^3^Department of Surgery, Randers Hospital, Randers, Denmark

**Keywords:** bile acid malabsorption, colonic epithelium, water transport, aquaporins, bile acid diarrhea

## Abstract

In normal individuals, the epithelium of the colon absorbs 1.5–2 l of water a day to generate dehydrated feces. However, in the condition of bile acid malabsorption (BAM), an excess of bile acids in the colon results in diarrhea. Several studies have attempted to address the mechanisms contributing to BAM induced by various bile acids. However, none have addressed a potential dysregulation of aquaporin (AQP) water channels, which are responsible for the majority of transcellular water transport in epithelial cells, as a contributing factor to the onset of diarrhea and the pathogenesis of BAM. In this study, we aimed to systematically analyze the expression of AQPs in colonic epithelia from rat, mouse, and human and determine whether their expression is altered in a rat model of BAM. Mass spectrometry-based proteomics, RT-PCR, and western blotting identified various AQPs in isolated colonic epithelial cells from rats (AQP1, 3, 4, 7, 8) and mice (AQP1, 4, 8). Several AQPs were also detected in human colon (AQP1, 3, 4, 7–9). Immunohistochemistry localized AQP1 to the apical plasma membrane of epithelial cells in the bottom of the crypts, whereas AQP3 (rat, human) and AQP4 (mice, human) were localized predominantly in the basolateral plasma membrane. AQP8 was localized intracellularly and at the apical plasma membrane of epithelial cells. Rats fed sodium cholate for 72 h had significantly increased fecal water content, suggesting development of BAM-associated diarrhea. Colonic epithelial cells isolated from this model had significantly altered levels of AQP3, 7, and 8, suggesting that these AQPs may be involved in the pathogenesis of bile acid-induced diarrhea.

## Introduction

Bile acids, synthesized and secreted by liver hepatocytes, are steroid acids that act as surfactants and aid solubilization, digestion, and absorption of lipids in the small intestine. The primary bile acids synthesized in the human liver are cholic acid (CA) and chenodeoxycholic acid (CDCA), which are conjugated to either taurine or glycine to form bile salts. Various modifications of bile acids confer different biological properties. Following secretion into the small intestine, 95% of bile acids are reabsorbed in the distal ileum *via* the apical ileal sodium-dependent bile acid cotransporter (ASBT, IBAT, or SLC10A2) (1). These bile acids are complexed to plasma proteins and recycled back to the liver *via* the enterohepatic circulation for further secretion into the biliary system and gallbladder. This process allows large amounts of bile acids to be secreted into the intestine, but a low rate of bile acid synthesis (2, 3). Despite this recycling, 400–800 mg of bile acids reach the colon every day. Here, they undergo microbial biotransformation to secondary bile acids, such as deoxycholic acid (DCA) and lithocholic acid (LCA) (2–4). DCA is the most prominent bile acid in the colon in humans (2). Different species have various bile acids, which constitute a “characteristic bile acid profile,” with CA found in bile of many mammalian species (5).

In addition to aiding lipid absorption, bile acids also have a wide range of other biological activities (5). For example, bile acids can regulate gene expression *via* various intracellular (nuclear) receptors, such as the farnesoid X receptor α (FXRα, NR1H4). FXRα activation is central in the regulation of bile acid production in the liver *via* a negative feedback system involving production of the ileal hormone fibroblast growth factor 19 (FGF19) (FGF15 in rodents) (6–9). Other intracellular receptors for bile acids include the vitamin D_3_ receptor (VDR, NR1I1), pregnane X receptor (PXR, NR1I2), and constitutive androstane receptor (CAR, NR1I3) (5, 10). Bile acids also bind to the plasma membrane-associated G-protein-coupled bile acid receptor 1 (TGR5, M-BAR, GPA, GPR131) stimulating cAMP production (11). Bile acid activation of this receptor stimulates the release of glucagon like peptide-1 (GLP1) from the enteroendocrine L cells of the small intestine, thus affecting glucose homeostatis. Locally, bile acids can affect colonic epithelial cells in a number of ways, e.g., increasing mucosal permeability and bacterial uptake (12), cell migration (13), apoptosis, and proliferation (14), and due to their antimicrobial activity, they contribute to regulation of the gut microbiome (4, 15), although not all processes have been described to be mediated by specific receptors. Furthermore, a fraction of the bile acids that returns to the liver *via* the portal vein escapes the transport into hepatocytes and thus reaches the systemic circulation (16). Due to the broad tissue localization of their receptors, bile acids are in principle capable of inducing effects outside the intestines, e.g., TGR5 is expressed in the brain, endocrine glands, and immune organs (17).

In conditions collectively referred to as bile acid malabsorption (BAM), an abundance of bile acids in the colon causes diarrhea (3). Although these excess bile acids often originate from diminished reabsorption due to various causes, e.g., ileal disease or ileal resection (3), a complete understanding of the mechanisms behind how excess bile acids induce diarrhea is lacking. High concentrations of bile acids in the colon decrease colonic absorption and increase secretion of electrolytes and water. These effects of bile acids, combined with the ability to increase colonic motility, are likely mediators of diarrhea (1, 18, 19). Although enhanced lubrication of the epithelia *via* increased mucus secretion accelerated colonic peristaltis (3), and potentially the ability of bile acids to serve as detergents have been proposed to play a role in bile acid-induced diarrhea, the most likely cause centers on bile acid-induced alterations in mucosal permeability. Several studies have suggested that bile acids activate CFTR and induce chloride secretion resulting in alterations in ionic gradients across colonic epithelial cells and enhanced water secretion (20–23). Whether this water secretion occurs *via* a claudin-mediated paracellular pathway or a transcellular pathway mediated by sodium-dependent cotransport mechanisms or one of the several aquaporin (AQP) water channels described to be present in the colon remains unknown (24, 25). Interestingly, it appears that pathophysiological levels of bile acids are required to induce secretory responses in epithelial cells (18), whereas physiological concentrations of bile acids may decrease colonic secretion (26). Determining the precise mechanism underlying the diarrhea observed in BAM is complicated further due to the variety of effects of individual bile acids. For example, bile acids can also induce morphological changes in the gut mucosa, such as rounding of the normally columnar epithelial cells, decreasing the height of the crypts of Lieberkühn in the colon, and shortening of the villi in the small intestine (27). Mucosal barrier function is also altered by changes in the composition and/or concentration of bile acids. For example, while CA, DCA, and CDCA cause increased intestinal permeability by altering tight junction proteins, ursodeoxycholic acid (UDCA) does not (28). Interestingly, while hydrophobic bile acids induce colonic epithelial cell apoptosis, co-administation with taurin-conjugated UDCA ameliorates the cytotoxicity of the hydrophobic bile acids (28).

Despite numerous studies linking colonic expression of AQPs with conditions of diarrhea (29, 30), a role for bile acids in regulating AQPs in the intestine is currently unknown. We hypothesized that various AQPs are present in colon epithelial cells and that their abundance is altered in an animal model of bile acid-induced diarrhea. Our studies indicate that AQPs have a heterogeneous expression pattern in rat, mouse, and human colonic epithelial cells. Furthermore, excess levels of bile acids can modulate expression of AQP3, AQP7, and AQP8, suggesting that these channels may be involved in the pathophysiology of bile acid-induced diarrhea.

## Materials and Methods

### Ethical Approval

All animal protocols comply with the European Community guidelines for the use of experimental animals. They were approved and performed under a license issued for the use of experimental animals by The Animal Experiments Inspectorate, Ministry of Food, Agriculture and Fisheries – Danish Veterinary and Food Administration (Dyreforsøgstilsynet) and methods performed in accordance with local guidelines and regulations. To determine the normal distribution of AQPs in human tissue, specimens of normal human tissue were obtained from fresh and healthy resection border of colon pieces that was removed due to cancer. Prior to surgery, all patients provided written permission to donate a colon sample for research after the surgical resection. All patient samples and data were anonymous and only age, gender, and date was reported at time of tissue isolation. This procedure was aligned with the Danish guidelines for collection of biological materials according to the Local Ethical Committee (Etisk Komite) (Act number 593 of 14 July, 2011, §2, number 1). A minimum of three donors were used.

### Rat Model of Bile Acid-Induced Diarrhea

Ten male Wistar Munich rats were initially housed in standard cages in a room kept at a constant temperature of 22°C with a 12:12-h light:dark cycle. Rats were fed a standard rodent chow (Altromin) and had water *ad libitum*. Animals were switched to rat metabolic cages housed in the same room. During an initial 3-day acclimatization period, the rats were fed standard rodent chow and had access to water *ad libitum*. Rats were randomly assigned to either a control group and fed a standard rodent chow or an experimental group that were fed standard rodent chow mixed with 1% weight/weight sodium cholate (Sigma). After an additional 3 days, rats were euthanized by cervical dislocation and colonic epithelial cells isolated using the Ca^2+^ chelation method as described below. Physiological parameters, including food and water intake, bodyweight, urine volume, and osmolality and feces output, were monitored on a daily basis. Feces water content was calculated by assessing the original wet weight of the feces relative to the weight of the feces after drying for 3 days at 60°C. Urine volume was measured gravimetrically assuming a density of 1, and osmolality was measured using freezing point depression (Advanced Instruments).

### Isolation of Rat or Mouse Colon Epithelia *via* Mucosal Scraping

Animals were housed in standard cages and fed standard rodent chow (Altromin) and had water *ad libitum*. Animals were euthanized by cervical dislocation. Colons were dissected and flushed repeatedly with PBS to clean off fecal matter. The colons were cut open and held at one end with a glass slide, while the colonic mucosa was scraped off gently using another glass slide. The scrapings were subjected to RNA extraction (see below).

### Isolation of Colonic Epithelia *via* Ca^2+^ Chelation

Epithelial cells were isolated using a protocol based on a previously published method (31). Briefly, colons were divided equally into a proximal and a distal region. After flushing extensively with PBS, the pieces of colons were inverted, filled with Ca^2+^-free Ringer solution (127 mM NaCl, 10 mM HEPES, 5 mM KCl, 5 mM Na-Pyruvate, 5 mM EDTA, 1 mM MgCl_2_, 5 mM glucose, pH 7.4), and ligated at each end. The pieces of colons were incubated in Ca^2+^-free Ringer solution for 20 mins at 37°C with constant shaking. The pieces of colon (mostly muscle and connective tissue) were removed and the epithelial cells pelleted by centrifugation at 4500 × *g* for 2 mins. Cell pellets were subjected to RNA extraction, or for protein analysis, the pellet was homogenized in dissection buffer (300 mM sucrose, 25 mM imidazole, 1 mM EDTA, pH 7.2) containing protease inhibitors leupeptin (1 mg/ml) and Pefa-block (0.1 mg/ml) (Boehringer Mannheim). SDS-PAGE gel samples were generated by addition of Laemmli sample buffer containing 10 mg/ml DTT.

### Isolation of Epithelia from Human Colonic Tissue

Also, 0.5 cm × 0.5 cm pieces of proximal and distal colon for immunohistochemistry were fixed by immersion fixation overnight at 4°C in 4% paraformaldehyde before embedding in paraffin. For protein and RNA preparations, tissue pieces were manually trimmed to enrich for mucosa. Tissue for RNA isolation was stored in RNAlater^®^ (Invitrogen) at 4°C for 24 h before RNA extraction using the RiboPure kit (Ambion) according to the manufacturer’s instructions. Protein samples were prepared as described above.

### Immunoblotting

Standard procedures were utilized for sample preparation and SDS-PAGE. Immunoblots were developed using ECL-detection and signal intensity in specific bands quantified using Image Studio Lite (Qiagen) densitometry analysis.

### Immunohistochemistry

Dissected pieces of colon were immersion fixed overnight at 4°C in 4% paraformaldehyde before embedding in paraffin. All procedures have been described in detail previously (32). Labeling was visualized by use of peroxidase-conjugated secondary antibodies for light microscopy (Dako, Glostrup, Denmark). Imaging was performed on a Leica DMRE light microscope with PL APO 63×/1.32–0.6 and PL Fluotar 25×/0.75 oil immersion objectives, and a Leica DC 300 digital camera. PAS staining was performed on sections by incubation in 1% periodic acid followed by rinsing and incubation with Schiff’s reagent. The sections were rinsed and incubated with Mayer’s Hematoxylin before mounting.

### PCR, RT-PCR, and Real-time Quantitative RT-PCR

RNA extraction was performed with the RiboPure kit (Ambion) according to the manufacturer’s instructions. All procedures, including determination of product specificity, have been described in detail previously (33). Primer pairs, which spanned an exon–exon junction, are detailed in Table 1.

**Table 1:** **Primer pairs used in the study**.

PCR primers		
Primer pair	Forward primer sequence 5′–3′	Reverse primer sequence 5′–3′
**Human**		
AQP1	GCCATCCTCTCAGGCATCAC	ACACCATCAGCCAGGTCATTG
AQP2	TTGGGATCCATTACACCGGC	TCCAGAAGACCCAGTGGTCA
AQP3	ACCAGCTTTTTGTTTCGGGC	GGCTGTGCCTATGAACTGGT
AQP4	GTGCTTTGGCCATATCAGCG	CACTGGGCTGCGATGTAGAA
AQP5	GCTCACTGGGTTTTCTGGGTA	CTTTGATGATGGCCACACGC
AQP6	TCGTAGGCTCCCACATCTCT	CTGTTCCGGACCACGTTGAT
AQP7	GGGGACACAGGGATAGCTGA	GTTTGCGTTCTTGGGGTGTC
AQP8 primer pair 1	CAGCCATGTCTGGTCGAACT	TGTCCACCACTGATATTCCCC
AQP8 primer pair 2	TTGGACTGCTCATTAGGTGCTT	AATGCAGGAACTCCCCTGTC
AQP9	GTGTCTCTGGTGGTCACATCA	AATGCCAAAGACGGTTGCAG
FXR	GGGTCTGCGGTTGAAGCTAT	GTCAGAATGCCCAGACGGAA
TGR5	TCAGCCAGGACACCAGACAT	AGGGTCCTTCCTGGGAGATGG
RNA polymerase II subunit RPB1	ACGCTGCTCTTCAACATCCA	GGCAGACACACCAGCATAGT
**Mouse**		
AQP1	ACCTGCTGGCGATTGACTAC	TGGTTTGAGAAGTTGCGGGT
AQP2	TGGCTGTCAATGCTCTCCAC	GGAGCAGCCGGTGAAATAGA
AQP3	TGCCTTGCGCTAGCTACTTT	GCCACAGCCAAACATCACAA
AQP4	ATTGGGAGTCACCACGGTTC	CGTTTGGAATCACAGCTGGC
AQP5	CGCTCAGCAACAACACAACA	CCGGTGAAGTAGATCCCCAC
AQP6	GGCCACCTCATTGGGATCTAC	ATCGCTGGGCTACAGTCTTG
AQP7	AACAAGTGTTCAGAGCCGGA	GATCCTGTGGTATGCTGGGG
AQP8	TGGTGAATGTCCCCAGTCCT	CATTGGTGTCTGCTCCCCAG
AQP9	GAAACTGAGCGAGCAGACCT	AGCCACATCCAAGGACAATCA
FXR	TGAGGGCTGCAAAGGTTTCT	CATACATTCAGCCAACATCCC
TGR5	GATGTACCCTCAACCCTGGC	ACAGAGTTCCAGGCCCTAGT
β-actin	ACATGGCATTGTTACCAACTGG	CGGACTCATCGTACTCCTGCTT
**Rat**		
AQP1	CCTGCTGGCCATTGACTACA	TGGTTTGAGAAGTTGCGGGT
AQP2	TTTCACCGGTTGCTCCATGA	GTCCGATCCAGAAGACCCAG
AQP3	AAGTGTCTGGAGCCCACTTG	CAGCTTGATCCAGGGCTCTC
AQP4	TGGACAGCTGTAAGTGTGGAC	ATGAGCATGGCCAGGAACTC
AQP5	CATGAACCCAGCCCGATCTT	AGAAGACCCAGTGAGAGGGG
AQP6	GGGCCATCTCATTGGGATTC	GCTACGGTCTTGGTGTCAGG
AQP7	GTTTGCGTTGTTGGGGTGTC	TTCCCGGCACTGAACACTTT
AQP8	AACATCAGCGGTGGACACTT	CCAGACGCATTCCAGAACCT
AQP9	CCGGATAGCGAAGGAGACAC	TGATGTGGCCCCCAGAGATA
FXR	GTGACAAAGAAGCCGCGAAT	TTCGGAAGAAACCTTTGCAGC
TGR5	CACTTGGCCCCCAACTTTTG	GGTAGGGGGCTGGGAAGATA
**Mouse and rat**		
Enteric smooth muscle actin 2	TGGACGGGATCTCACAGACTAC	ACAATTTCTCTCTCAGCTGTGGTCA
Collagen type I α 1	GAGAGGTGAACAAGGTCCCG	AAACCTCTCTCGCCTCTTGC
**Human, mouse, and rat**		
18S rRNA	GGATCCATTGGAGGGCAAGT	ACGAGCTTTTTAACTGCAGCAA

### Reverse-Phase Liquid Chromatography Fractionation, Nano-Liquid Chromatography, and Mass Spectrometry Analysis

Epithelial cell samples from rat distal or proximal colon were reduced, alkylated, and digested using trypsin and desalted using C18 columns (Waters) prior to further fractionation as previously described (34). The peptides were separated by high-pH RPLC using a Dionex Ultimate 3000 LC system (Thermo Scientific) with a ZORBAX Extended-C18 LC column (2.1 mm × 150 mm, 5 μm, Agilent). Buffer A (25 mM NH_4_FA in 100% H_2_O, pH = 10) and B (25 mM NH_4_FA in 90% ACN, pH = 10) were used for gradient separation. The gradient was 0–10% B (0–10 min), 10–35% B (10–50 min), and 35–80% B (50–64 min), with 32 fractions collected every 2 min. The 32 fractions were further pooled into 8 by mixing equal-time-interval fractions, for example, fraction 1, 9, 17, and 25 were mixed together. The resulting eight fractions were lyophilized in a SpeedVac, and then resuspended in 0.1% FA for LC–Mass Spectrometry (MS)/MS analysis. Analysis was by nano-liquid chromatography (nLC) (EASY-nLC 1000, Thermo Scientific) coupled to a mass spectrometer (Q Exactive, Thermo Fisher Scientific) through an EASY-Spray nano-electrospray ion source (Thermo Scientific). A pre-column (Acclaim^®^PepMap 100, 75 μm × 2 cm, nanoviper fitting, C18, 3 μm, 100 Å, Thermo Scientific) and analytical column (EASY-Spray Column, PepMap, 75 μm × 15 cm, nanoviper fitting, C18, 3 μm, 100 Å, Thermo Scientific) were used to trap and separate peptides, respectively. For nLC separation, buffer A was 0.1% FA and buffer B was 95% ACN/0.1% FA. A 30-min gradient of 1 to 35% buffer B was used for peptide separation. MS constituted of full scans (m/z 300–1800) at a resolution of 70,000 (at m/z 200) followed by up to 10 data-dependent MS/MS scans at a resolution of 17,500. HCD collision energy was 28%. Dynamic exclusion of 30 s as well as rejection of precursor ions with charge state +1 and above +8 was employed.

### MS Data Analysis and Data Inclusion Criteria

Raw files were, respectively, searched against rat and mouse protein databases (rat RefSeq database downloaded August 2015 containing 42,925 sequences, mouse RefSeq database downloaded October 2014 containing 58,513 sequences) using the SEQUEST algorithm embedded in Proteome Discoverer (PD) software (Thermo Scientific, version 1.4). Precursor mass tolerance was set as 10 ppm, and fragment mass tolerance was set as 0.02 Da. Number of maximum miss cleavage sites was set to 2. Carbamidomethylation of cysteine was set as static modification. N-terminal acetylation, methionine oxidation, as well as phosphorylation of serine, threonine, and tyrosine were included as variable modifications. False discovery rate (FDR) was calculated using Percolator. Only rank 1 and high confidence (with a target FDR *q*-value below 0.01) peptides were included in the final results. Proteome GO-term molecular function analysis was performed using the Automated Bioinformatics Extractor^1^ (ABE) (35).

### Antibodies for Immunohistochemistry and Western Blotting

Rabbit anti-AQP1 was originally characterized by Nielsen et al. (36), including preadsorption controls, and has been used in multiple publications and knockout mice (37–39). Rabbit polyclonal anti-AQP3 (ab135694, Abcam) is a commercial antibody. Rabbit anti-AQP3 (8249) has been characterized previously (40, 41). Rabbit anti-AQP4, 249-323 (Alomone) has been used in a number of publications, e.g., Ref. (42). Rabbit anti-AQP7, 1246, has been characterized with the use of knockout animals (43, 44). Mouse monoclonal anti-AQP8 (ab77198, Abcam) is a commercial antibody. Rabbit anti-AQP8 1262 has been characterized previously with preadsorption controls for western blotting and immunohistochemistry in rat samples (45). Rabbit anti-actin A2066 was purchased from Sigma.

### Statistics

Statistical significance of qPCR data was determined using the Relative Expression Software Tool V2.0.13 (REST 2009) with 4000 randomizations (46). Relative changes in RNA expression between groups was performed as described (47). For each animal treatment group, *n* = 5. Technical replicates were performed on each cDNA sample for qPCR. Other data were tested for normal distribution using the D’Agostino–Pearson omnibus test and Graphpad Prism Software. Data fitting a normal distribution were analyzed using multiple *t*-tests, and statistical significance (*P* < 0.05) was determined using the Holm–Sidak method. All data are presented as mean ± SE.

## Results

### Isolation of Colonic Epithelial Cells

**Figure 1:**
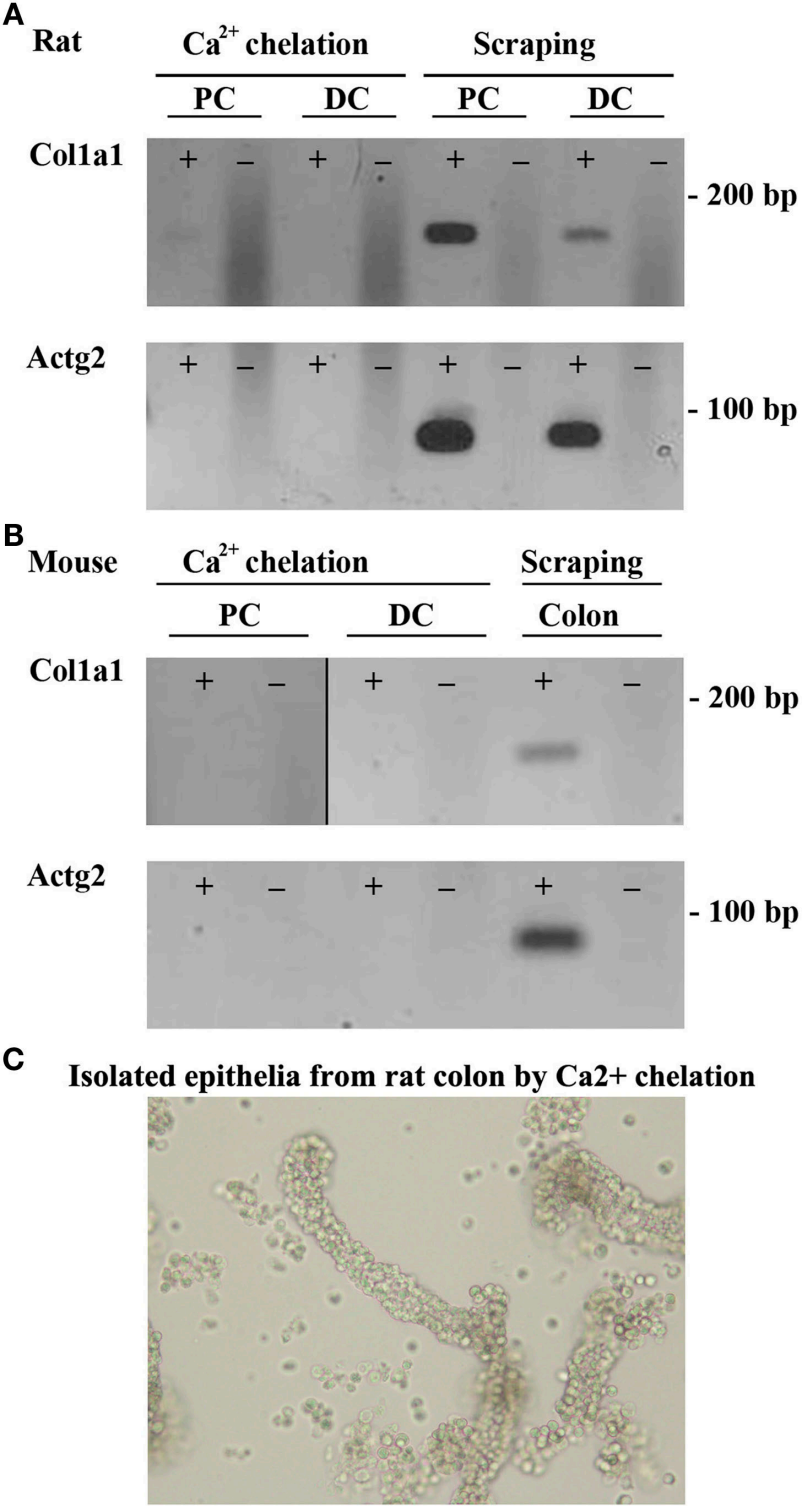
**Ca^2+^ chelation provides a pure population of colonic epithelial cells**. RT-PCR of colonic epithelial cells from rat **(A)** or mouse **(B)** isolated using Ca^2+^ chelation or mucosal scrapings using a marker of connective tissue, Col1a1 (alpha-1 type I collagen), or a marker of smooth muscle, Actg2 (actin, gamma 2, smooth muscle, enteric). **(C)** Brightfield microscopy of rat samples from Ca^2+^ chelation showing purification of both single cells and whole crypts. ± indicates reverse transcriptase enzyme included in RT reaction. PC, proximal colon; DC, distal colon.

Two methods to isolate colonic epithelia are mucosal scraping (29) or Ca^2+^ chelation (31). To determine which procedure provided the most pure population of epithelial cells, RT-PCR was used to compare the mRNA levels of alpha-1 type I collagen (Col1a1), a marker for connective tissue, and actin gamma 2 (Actg2), a marker of smooth muscle in samples isolated from both rats and mice using the two techniques. In contrast to mucosal scrapings, in epithelial cell samples prepared by Ca^2+^ chelation, Col1a1 and Actg2 were undetectable (Figures 1A,B). Brightfield microscopy of samples isolated by Ca^2+^ chelation revealed single cells and full length crypts (Figure 1C) further demonstrating their purity. Additional isolations from other animals had similar results, demonstrating the reproducibility of the technique. The remaining experiments were thus performed on samples prepared by the Ca^2+^ chelation method.

### Proteome of Rat and Mouse Colonic Epithelial Cells

**Figure 2:**
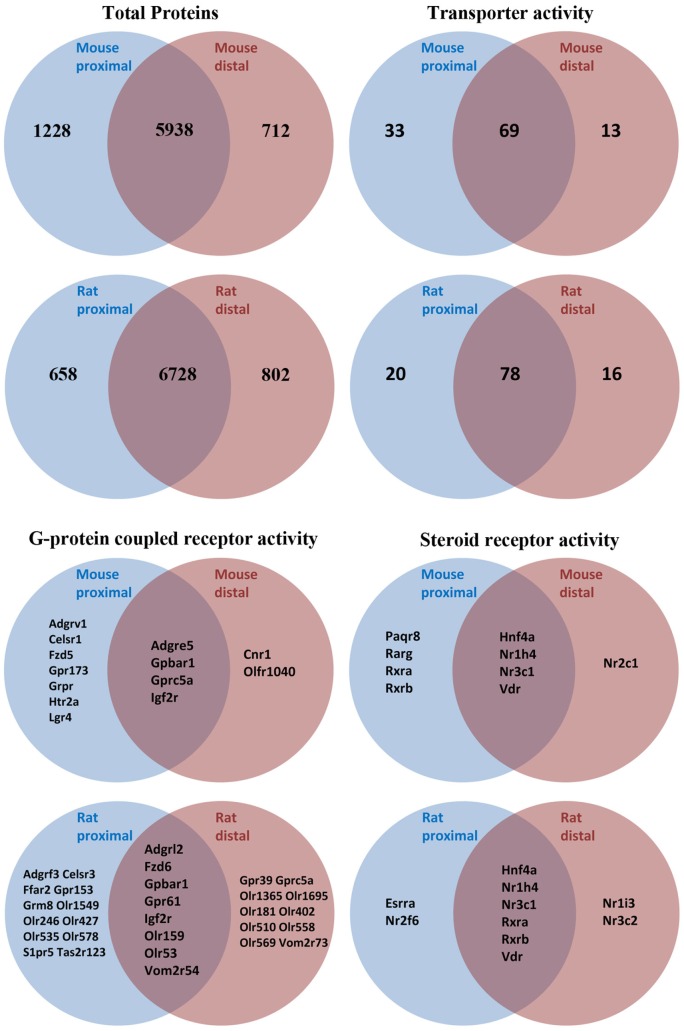
**Summary of proteomic data and GO-term analysis of rat and mouse colonic epithelial cells**. Only high confidence identifications (minimum of two unique peptides identified per protein) were used to segregate between expression in proximal or distal colon.

As an initial screen to identify AQPs and other potential modulators of bile acid signaling in colonic epithelia, proteomic profiling of purified epithelial cells from mouse or rat distal and proximal colon was performed using LC-ESI MS/MS. In mouse, unique peptides corresponding to 6563 proteins were identified from proximal colon samples and 5746 proteins from distal colon samples (Figure 2 and Data Sheet S1 in Supplementary Material). Together, this relates to 7878 unique proteins in mouse colon epithelial cells. Analysis of only high confidence identifications (minimum of 2 unique peptides identified per protein) suggested that 712 proteins were unique to distal colon, with 1228 unique to proximal colon. In rat proximal colon samples, 6561 proteins were identified and 6737 from distal colon samples (Figure 2 and Data Sheet S1 in Supplementary Material), thus making a total of 8188 unique proteins in rat colon epithelial cells. Analysis of only high confidence identifications suggested 802 proteins were unique to distal colon, with 658 unique to proximal colon. All proteins identified (including protein accession number, gene symbol, and number of peptide identifications) are available online.^2^^,^^3^^,^^4^^,^^5^

Proteome GO-term molecular function analysis using Panther (48) highlighted a number of major processes and protein classes highly enriched in rat and mouse colonic epithelial cells (Data Sheets S2 and S3 in Supplementary Material). Additional GO-term analysis in mouse (Figure 2) highlighted 115 proteins with known transporter activity (including AQP1, AQP2, and AQP4), 2 adenylate cyclase isoforms, 9 steroid receptors (including the FXR and VDR), and 18 GPCRs (including the G-protein-coupled bile acid receptor 1). Further analysis of the mouse colon proteome versus published databases (49) identified multiple E1 and E2 enzymes of the ubiquitin/sumo conjugation cascade, in addition to at least 212 E3 ligases and 44 proteins with known deubiquitylation activity. Similar GO-term analysis in rat (Figure 2) highlighted 114 proteins with known transporter activity (including AQP3 and AQP8), 3 adenylate cyclase isoforms, 10 steroid receptors (including the FXR and VDR), and 36 GPCRs (including the G-protein-coupled bile acid receptor 1).

### Targeted Identification of AQPs in Mouse, Rat, and Human Colon Samples

**Figure 3:**
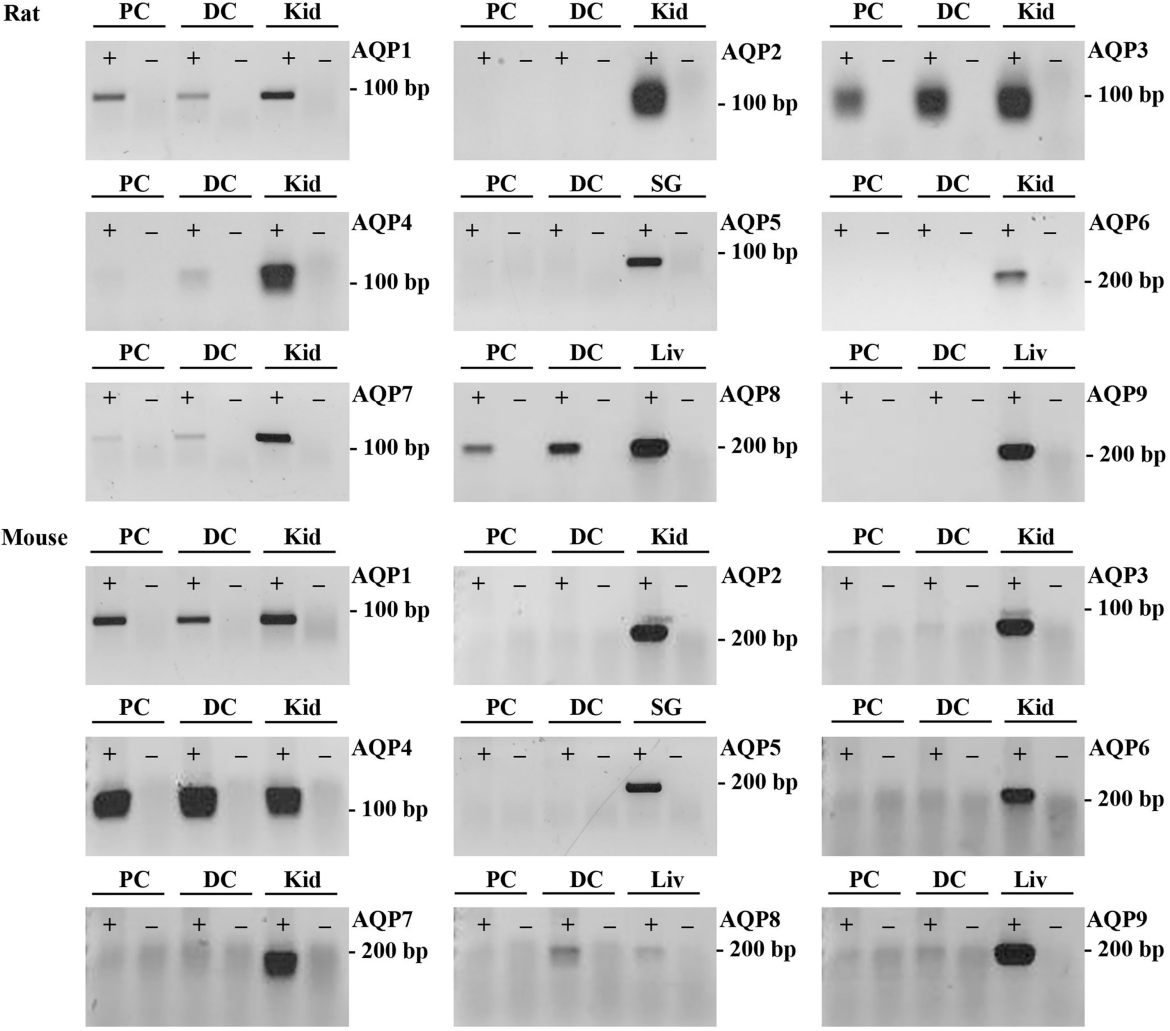
**RT-PCR to determine the expression of AQP1–9 in rat and mouse colonic epithelial cells isolated by Ca^2+^ chelation**. ± indicates reverse transcriptase enzyme included in RT reaction. PC, proximal colon; DC, distal colon; Kid, kidney; SG, salivary gland; Liv, liver.

**Figure 4:**
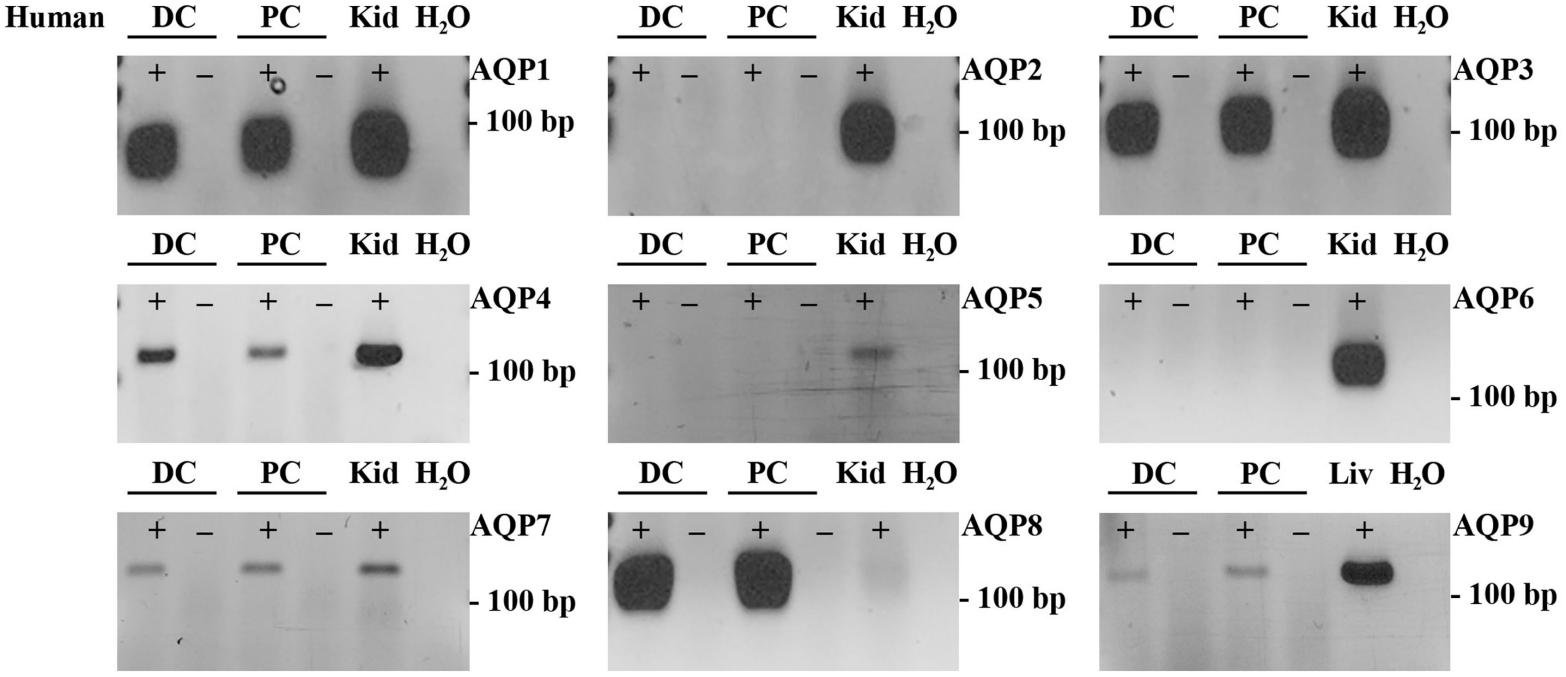
**RT-PCR to determine the expression of AQP1–9 in human colon biopsies**. ± indicates reverse transcriptase enzyme included in RT reaction. PC, proximal colon; DC, distal colon; Kid, kidney; Liv, liver.

**Figure 5:**
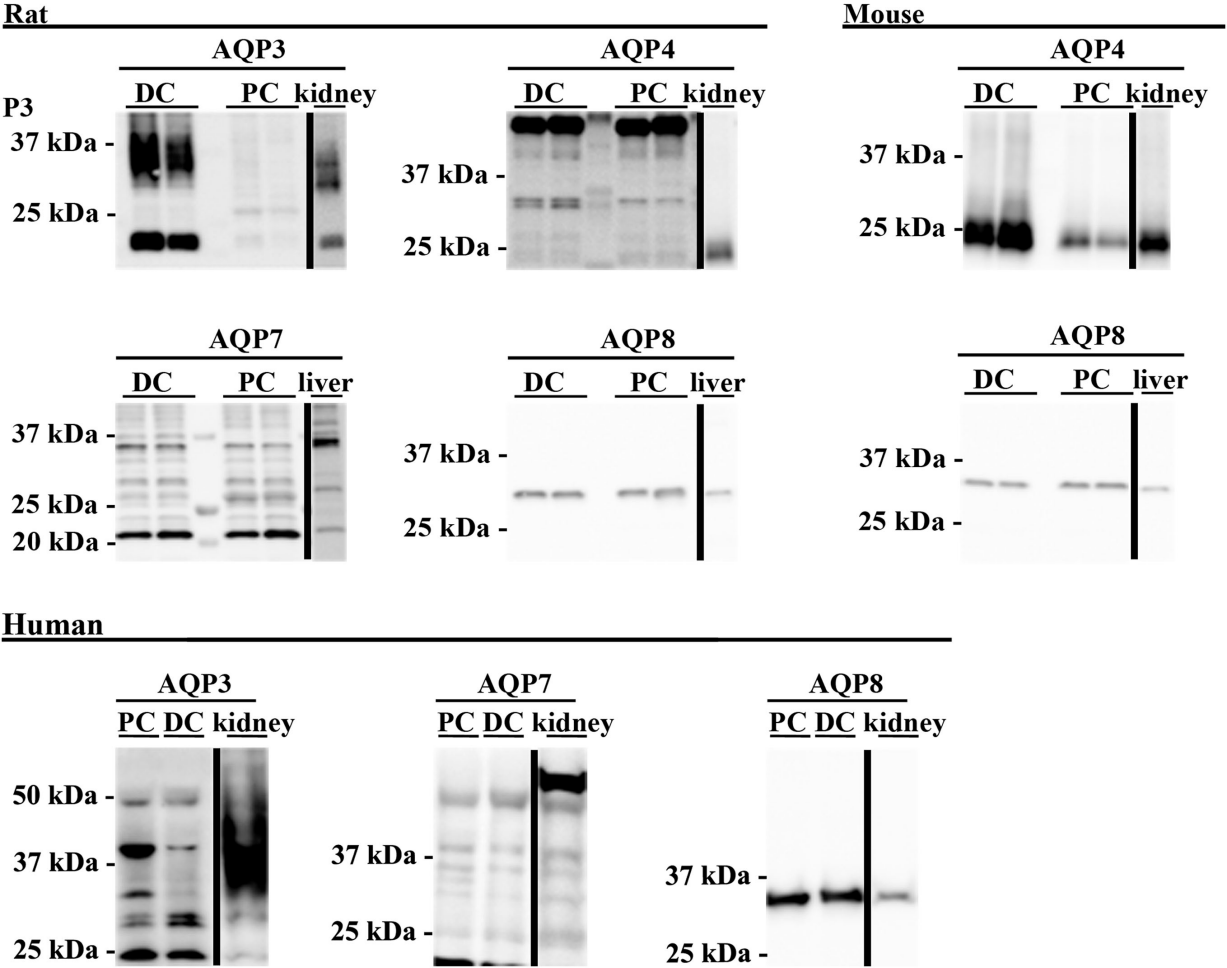
**Western blotting was used to assess expression of AQPs at the protein level**. Western blotting of colon epithelial cells purified by Ca^2+^ chelation from two male Wistar rats and four female mice. Human samples were not prepared by Ca^2+^ chelation. PC, proximal colon; DC, distal colon.

To supplement the identification of AQPs in rat and mouse colonic epithelial cells using protein MS, a more sensitive RT-PCR approach was utilized to specifically identify whether AQP1–9 were expressed in rat- and mouse-isolated colonic epithelial cells (Figure 3). In rats, AQP1, 3, 4, 7, and 8 were expressed, whereas in mouse AQP1, 4, and 8 were identified. In human colon biopsies, AQP1, 3, 4, 7, 8, and 9 were identified by RT-PCR (Figure 4). Expression of AQP3, 4, 7, and 8 were confirmed in rat colonic epithelial cell samples by western blotting, alongside AQP4 and AQP8 in mouse samples (Figure 5). In human samples, we confirmed expression of AQP3, 7, and 8 (Figure 5).

### Immunohistochemical Analysis of AQPs in Rat, Mouse, and Human Colon

Aquaporins identified at the mRNA level were examined by immunohistochemistry using specific antibodies to confirm their expression at the protein level in epithelial cells and to determine their subcellular localization.

#### AQP1

**Figure 6:**
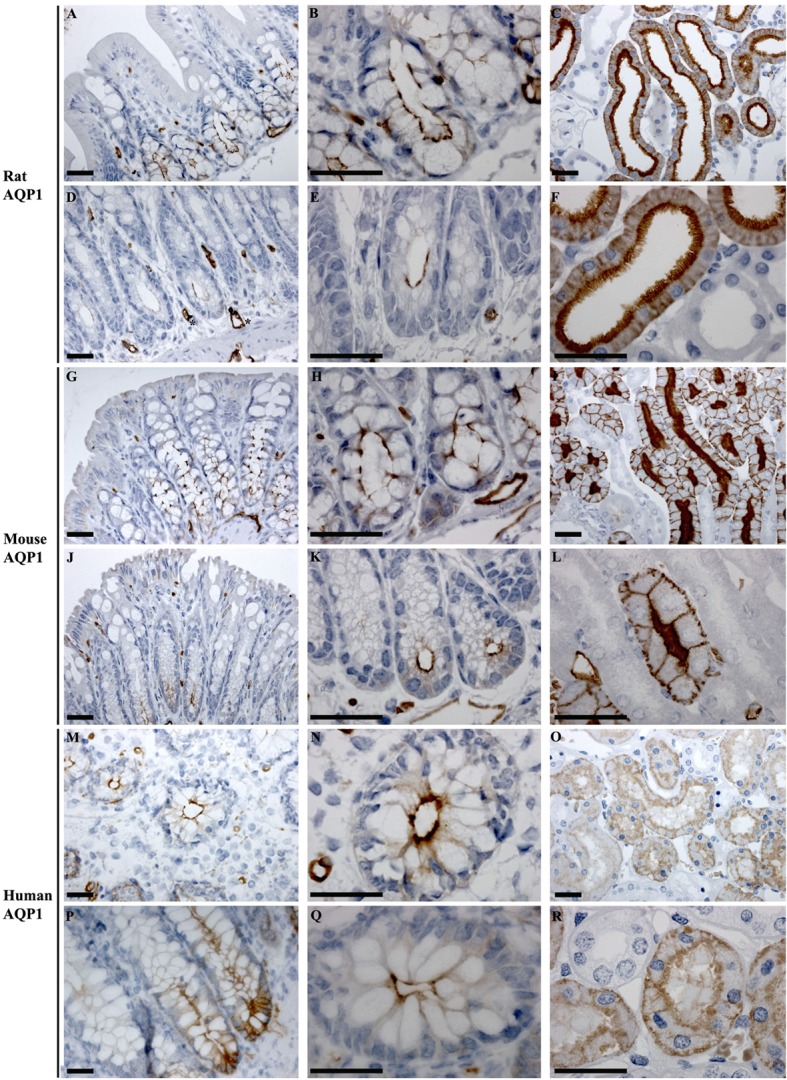
**Immunohistochemistry of AQP1 in rat, mouse, and human colon sections**. **(A,B)** Proximal rat colon. **(D,E)** Distal rat colon. **(C–F)** Rat kidney. **(G,H)** Proximal mouse colon. **(J,K)** Distal mouse colon. **(I–L)** Mouse kidney. **(M,N)** Proximal human colon. **(P,Q)** Distal human colon. **(O–R)** Human kidney. Scalebar = 100 μm. *indicates labeling of endothelial cells lining vasculature in the connective tissue.

In rat proximal and distal colon, positive staining of AQP1 was observed in the apical pole of epithelial cells at the base of the crypts (Figures 6A,B,D,E). Strong AQP1 immunoreactivity was also detected in endothelial cells (stars Figure 6D), known for their high AQP1 expression levels (50, 51). In mouse colon (Figures 6G,H,J,K), in addition to apical labeling of epithelial cells at the base of crypts, there was also cytoplasmic vesicular-like labeling on the apical side of the nucleus in surface epithelial cells. In human proximal and distal colon, AQP1 was localized to the apical membrane of epithelial cells at the base of crypts in (Figures 6M,N,P,Q), with some areas demonstrating the appearance of basolateral staining. Labeling of AQP1 in the kidney proximal tubule brush border and basolateral membrane (Figures 6C,F,I,L,O,R) confirmed antibody specificity (36, 52).

#### AQP3

**Figure 7:**
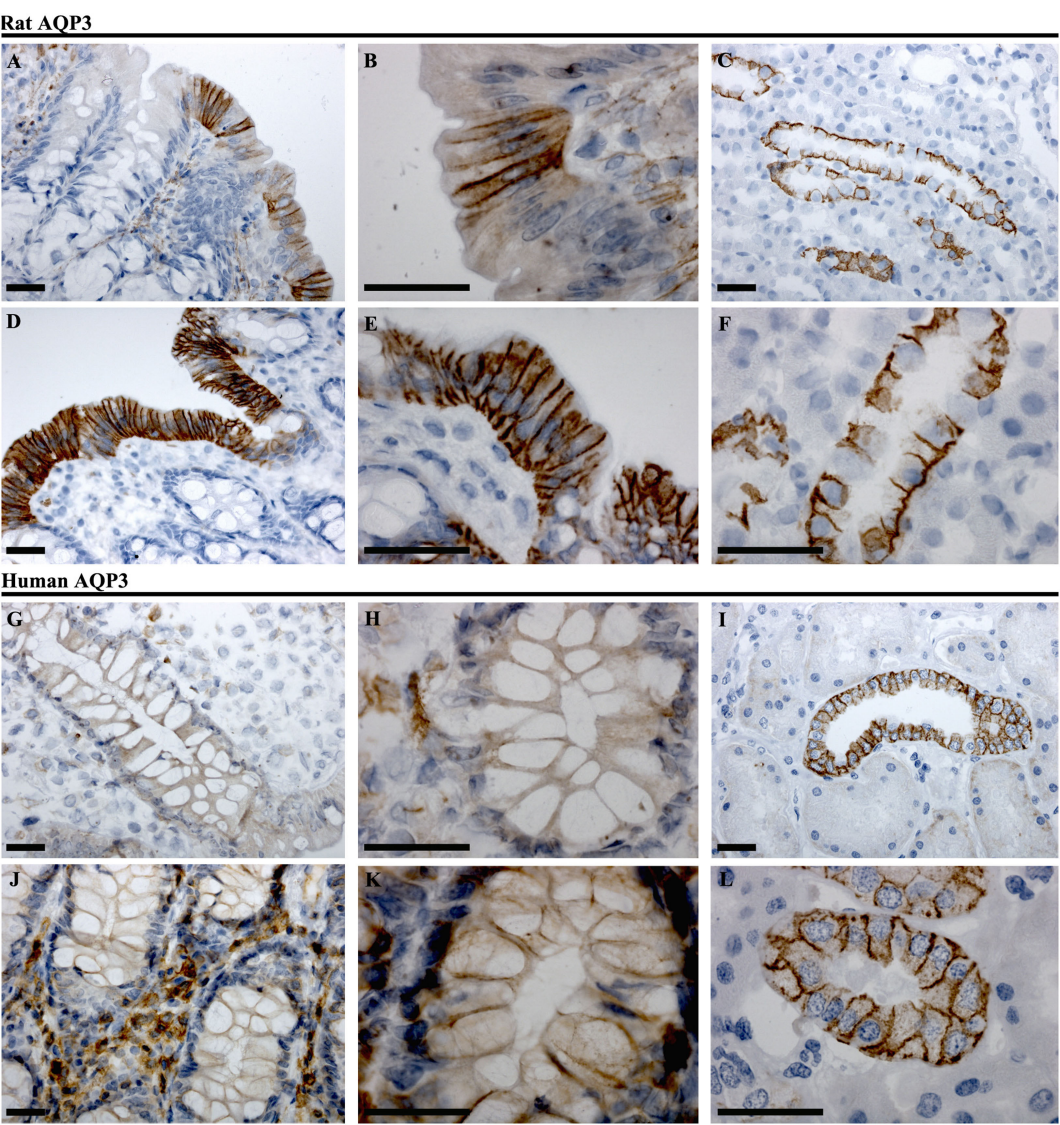
**Immunohistochemistry of AQP3 in rat and human colon sections**. **(A,B)** Proximal rat colon. **(D,E)** Distal rat colon. **(C–F)** Rat kidney. **(G,H)** Proximal human colon. **(J,K)** Distal human colon. **(I–L)** Human kidney. Scalebar = 100 μm.

In rat proximal colon, AQP3 was localized to the basolateral membrane of a subsection of surface epithelial cells (Figures 7A,B). The surface epithelial cells expressing AQP3 were localized in “patches,” with a gradual increase in the number of labeled cells from proximal to distal colon. In the distal colon, AQP3 was abundant in the basolateral membrane of nearly all surface epithelial cells (Figures 7D,E). Strong staining of the kidney collecting duct principal cells’ basolateral plasma membranes using this antibody confirmed the specificity of the staining (Figures 7C,F). In human colon sections, the most prominent AQP3 labeling was in cells in the distal part of colon, but in the connective tissue (lamina propria) rather than in epithelial cells (Figures 7G,H,J,K). Weak lateral labeling of AQP3 could be detected among some cells’ lining the crypts in the distal colon. Positive and specific staining of the human kidney collecting duct principal cell basolateral plasma membranes confirmed antibody specificity (Figures 7I,L).

#### AQP4

**Figure 8:**
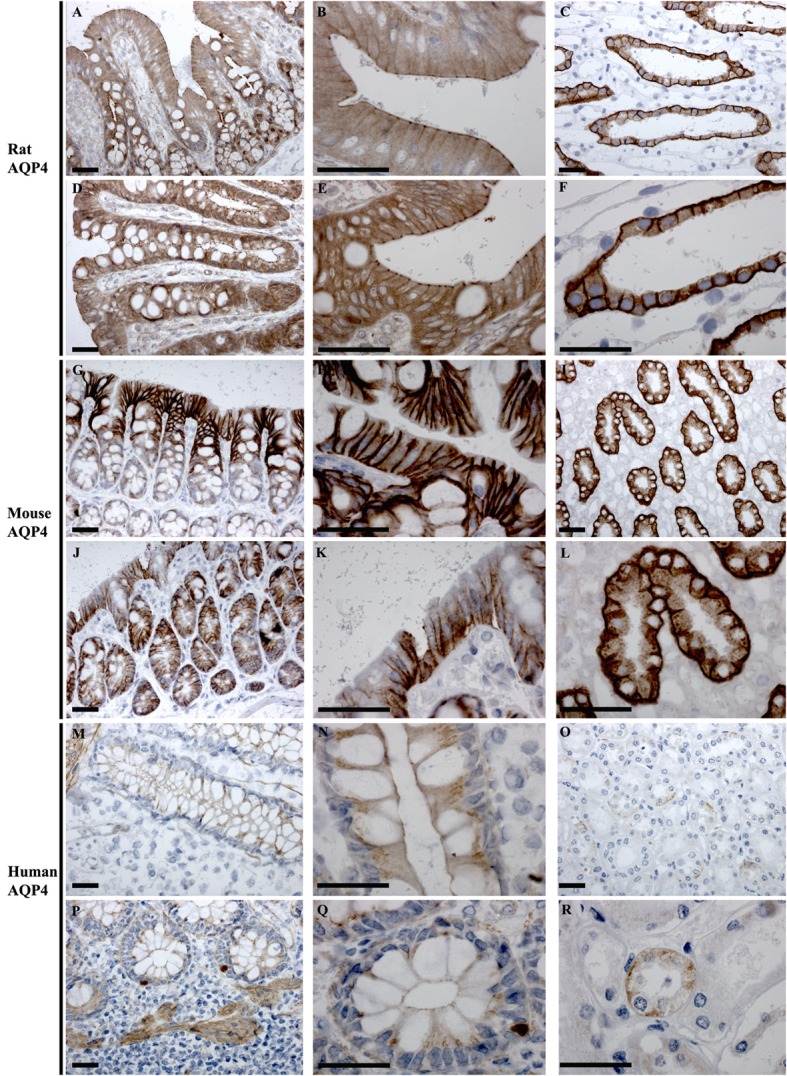
**Immunohistochemistry of AQP4 in rat, mouse, and human colon sections**. **(A,B)** Proximal rat colon. **(D,E)** Distal rat colon. **(C–F)** Rat kidney. **(G,H)** Proximal mouse colon. **(J,K)** Distal mouse colon. **(I–L)** Mouse kidney. **(M,N)** Proximal human colon. **(P,Q)** Distal human colon. **(O–R)** Human kidney. Scalebar = 100 μm.

In rat, AQP4 mRNA levels determined by RT-PCR were low (Figure 3). Immunolabeling of AQP4 in rat proximal and distal colon revealed a distinct labeling toward the apical pole of epithelial cells, in the regions corresponding to immediately below the tight junctions. This labeling was apparent on surface epithelia and in crypts (Figures 8A,B,D,E). Less abundant labeling of AQP4 was observed in the basolateral membrane. Some staining of smooth muscle was observed (not shown). Abundant labeling of basolateral membranes of kidney collecting duct principal cells confirmed specificity of the antibody (Figures 8C,F). In mouse proximal and distal colon, abundant AQP4 labeling was observed in the basolateral membrane of surface epithelial cells, which also extended into crypts (Figures 8G,H,J,K). In human, AQP4 labeling of the epithelial cells was generally weak (Figure 8). However, occasional strong labeling of single cells in the crypts of the epithelia with an intracellular and basal staining (subnuclear) was observed (Figures 8P,Q). AQP4 was also detected in smooth muscular layers.

#### AQP7

AQP7 was detected at low levels by RT-PCR in rat and human colon samples. However, antibodies that reliably detected AQP7 in kidney sections, where it is localized to the brush border of the proximal tubules (43), were not available. Thus immunohistochemical analysis of AQP7 in colon was not performed.

#### AQP8

**Figure 9:**
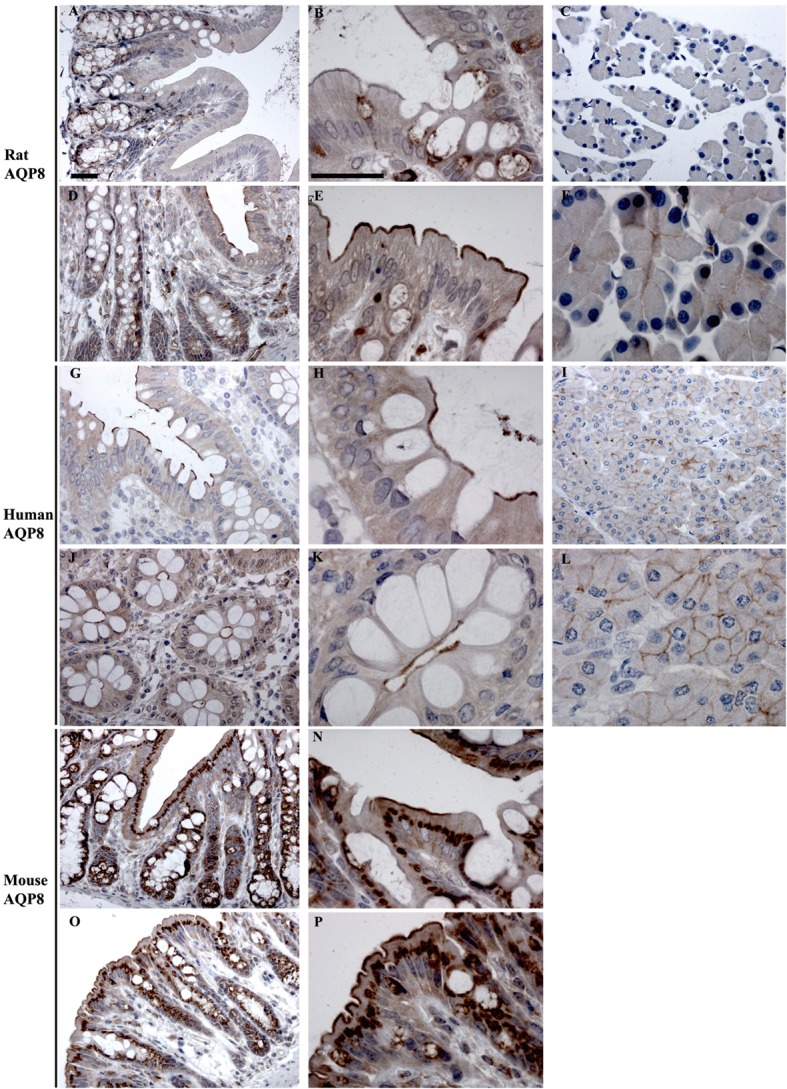
**Immunohistochemistry of AQP8 in rat, human, and mouse colon sections**. **(A,B)** Proximal rat colon. **(D,E)** Distal rat colon. **(C–F)** Rat pancreas. **(G,H)** Proximal human colon. **(J,K)** Distal human colon. **(I–L)** Human pancreas. **(M,N)** Proximal mouse colon. **(O,P)** Distal mouse colon. Scalebar = 100 μm.

In rats and human, AQP8 was abundantly detected in colon by RT-PCR, whereas it was of low expression levels in mouse colon (Figure 3). Immunohistochemistry of AQP8 demonstrated positive immunolabeling of proximal and distal colon, with more prominent staining of the distal colon. Labeling was abundant in the apical brush border of surface epithelial cells, with additional labeling of supra nuclear vesicles in the surface and crypt epithelia (Figures 9A,B,D,E). Labeling of the apical pole of pancreatic acinar cells (53, 54) confirmed specificity of the antibody (Figures 9C,F). In humans, labeling of AQP8 was apparent at the apical brush border of epithelial cells in both proximal and distal colon (Figure 9). Labeling of the apical pole of pancreatic acinar cells (55) confirmed specificity of the antibody in humans (Figures 9I,L). In mouse, a similar distribution of AQP8 at the apical surface of both proximal and distal colon epithelial cells, alongside intracellular labeling of distinct structures (possibly mitochondria), was observed using high concentrations of antibody (Figures 9M,P). However, a lack of consistent labeling of AQP8 in mouse liver or pancreas indicates that the immunohistochemical localization of AQP8 presented here must be interpreted with caution.

#### AQP9

In human colon, AQP9 could be detected by RT-PCR (Figure 3). Using a variety of different AQP9 antibodies, positive labeling of plasma membranes within hepatocytes was observed (56). However, a lack of consistent results using these antibodies on human colon sections (not shown), including diverse labeling of various cell types, prevents solid conclusions to be drawn regarding the localization of AQP9 in human colon epithelial cells.

### Feeding of Bile Acids Induces Diarrhea in Rats

**Figure 10:**
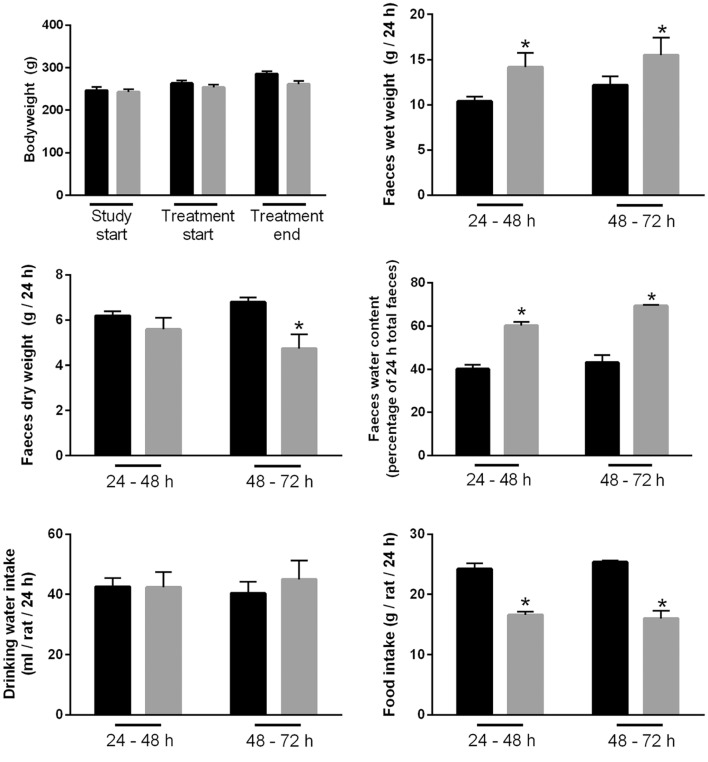

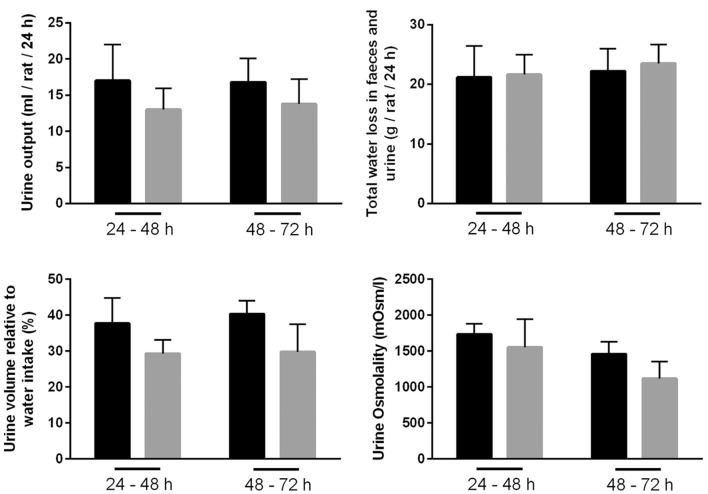
**Physiological parameters of either controls or rats treated with bile acids**. Time points indicate time elapsed after shift in dietary intake (see Materials and Methods).

**Figure 11:**
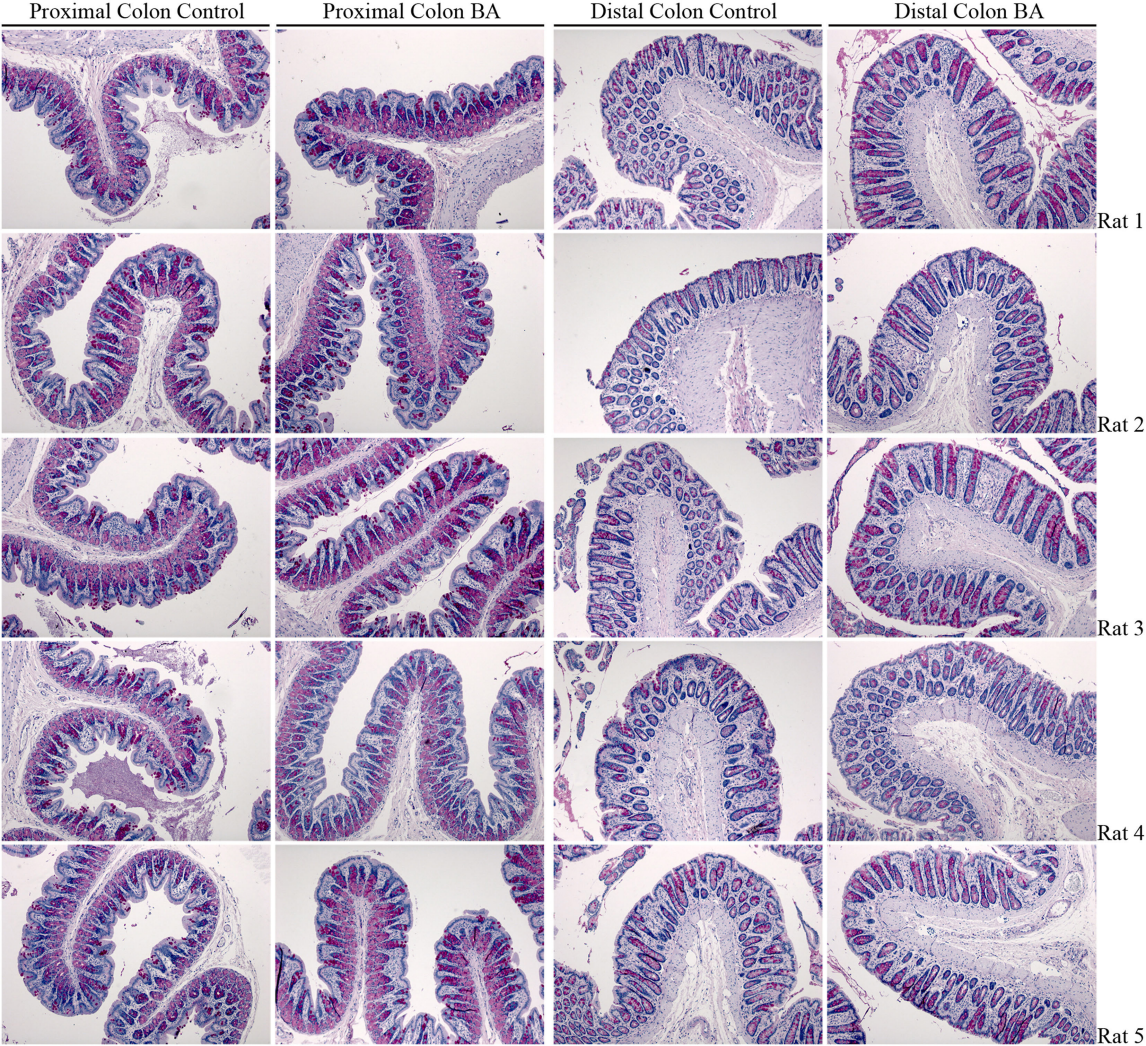
**PAS staining of rat colon isolated from control- and bile acid-treated rats**.

A rat model of BAM was generated by feeding animals 1% sodium cholate mixed in standard rodent chow for 3 days. Animals were housed in metabolic cages throughout the acclimatization and experimental period in order to collect physiological information regarding their response to treatment compared to control animals receiving standard rodent chow. Within 24 h, sodium cholate-fed rats developed a mild diarrhea, as demonstrated by significantly increased wet weight of stools and increased water content (Figure 10). Similar observations were apparent up until 72 h. The rats partially compensated for this additional water loss in the stools by reducing their urine output, and overall water loss in stools and urine was not significantly different between the groups. Sodium cholate-fed animals ate significantly less food, but their bodyweight after 72 h was not significantly different from controls. Together, the physiological data indicated that feeding rats additional bile acids in their diet was successful for creating a model of BAM resulting in diarrhea. PAS staining of the distal and proximal colon demonstrated no obvious gross morphological differences in the colonic mucosa from control- and bile acid-treated groups (Figure 11).

### Altered mRNA Expression of AQP3, 7, and 8 in Colonic Epithelial Cells Isolated from Bile Acid-Fed Rats

**Figure 12:**
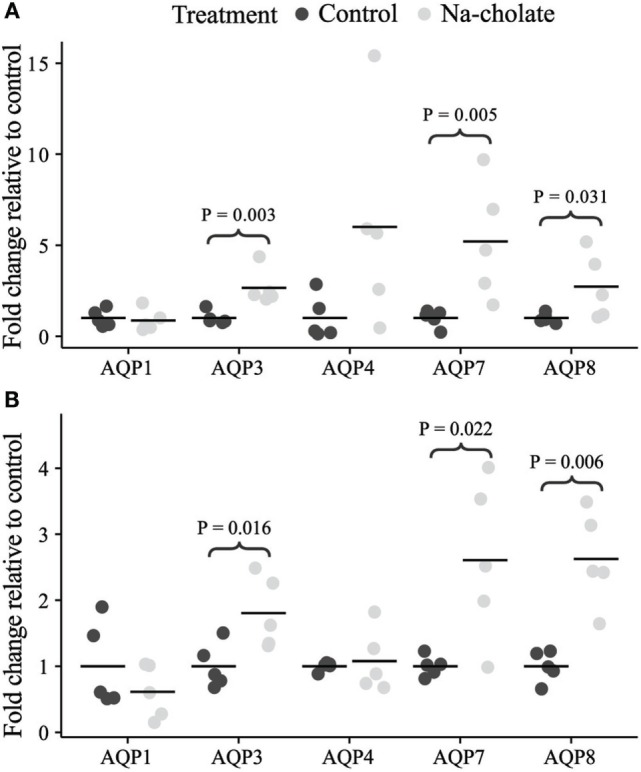
**mRNA expression levels of aquaporins in controls or rats treated with bile acids for 72 h**. **(A)** Proximal colon. **(B)** Distal colon.

**Figure 13:**
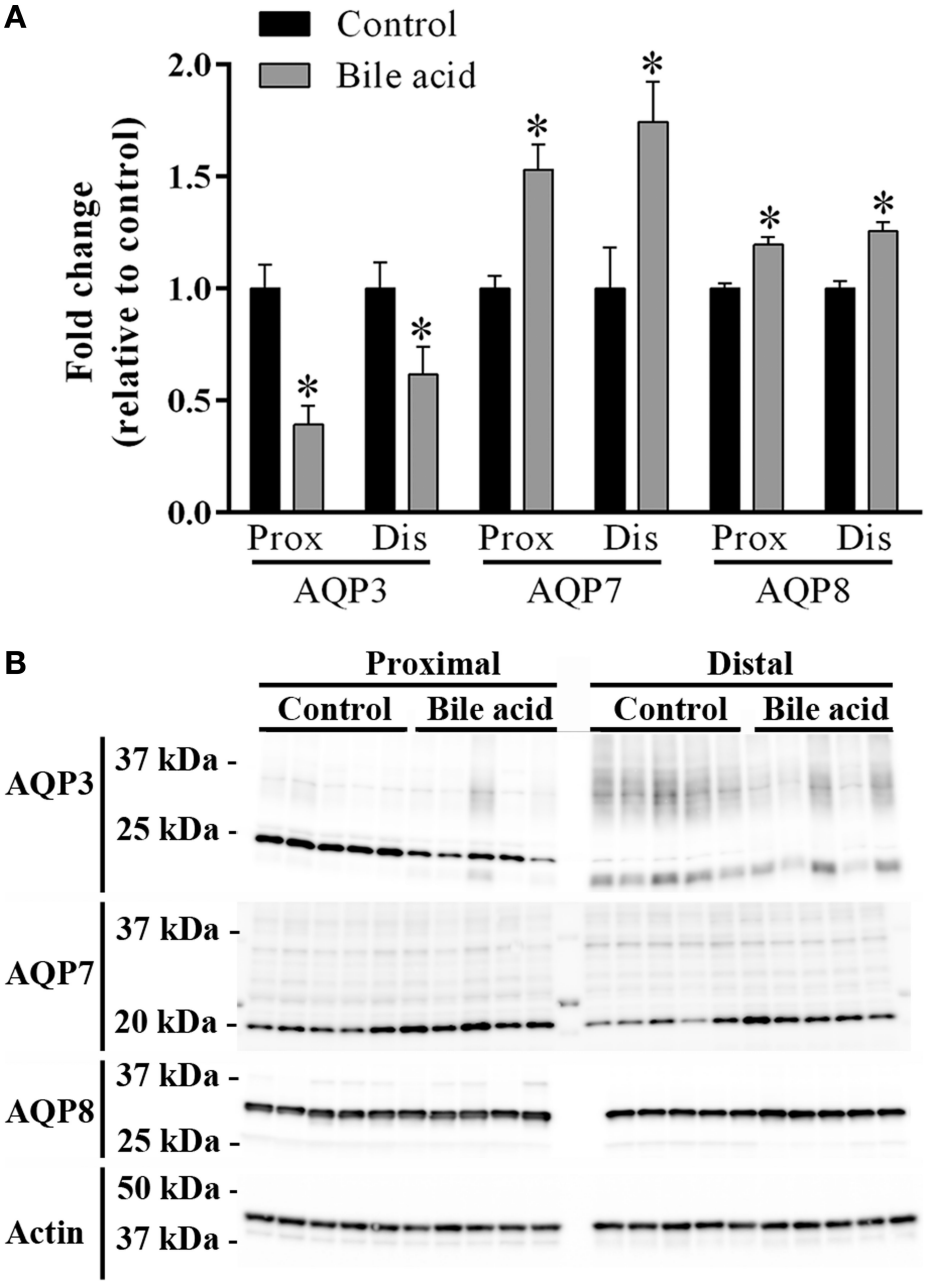
**Protein changes of AQP3, AQP7, and AQP8 in control rats versus rats treated with bile acids**. **(A)** Quantification of western blotting from the animal experiment. **(B)** Western blots corresponding to the quantification. Actin is shown as loading control. *indicates significant difference to control, *P* < 0.05%.

Initially, standard RT-PCR was used to examine if AQPs not detectable in rat colonic epithelial cells under basal conditions would increase in abundance to a detectable level following treatment of rats with bile acids. However, only the AQPs originally expressed under basal conditions were detected (data not shown). Subsequently, RT-qPCR was used to measure changes in the expression of AQP1, 3, 4, 7, and 8 mRNAs in the colonic epithelial cells isolated from both the proximal and the distal colon. Significant increases in AQP3, 7, and 8 were detected in the bile acid-treated group compared to the control group (Figure 12). In parallel, the protein levels of AQP7 and AQP8 were increased in the bile acid-treated group (Figures 13A,B), but in contrast to mRNA levels, AQP3 protein levels decreased in the treated group compared to the control group. Examination of colon tissue using immunohistochemistry did not reveal clear differences in the cellular or subcellular distribution of AQP3 or AQP8 following bile acid treatment (data not shown). Pearson product–moment correlation coefficients of AQP colonic abundance (western blotting level) and the measured physiological parameters showed various significant associations (Table 2). For example, there was a significant correlation between feces water content and analyzed AQPs (with the exception of AQP7 in the proximal colon). Spearman’s rank correlation coefficients confirmed a significant correlation between feces water content and AQP expression (data not shown).

**Table 2:** **Pearson product–moment correlation coefficients of various physiological parameters from metabolic cage studies and AQP expression in rat colon**.

	Proximal AQP3		Distal AQP3		Proximal AQP7		Distal AQP7		Proximal AQP8		Distal AQP8	
	Pearson *r*	*P* values	Pearson *r*	*P* values	Pearson *r*	*P* values	Pearson *r*	*P* values	Pearson *r*	*P* values	Pearson *r*	*P* values
Feces wet weight (output/g per g BW)	−0.798	0.010	−0.720	0.029	0.299	0.434	0.748	0.021	0.668	0.049	0.488	0.183
Feces dry weight (output/g per g BW)	0.414	0.268	0.273	0.477	−0.752	0.019	−0.381	0.312	−0.477	0.194	−0.759	0.018
Feces water content (g per g BW)	−0.893	0.001	−0.775	0.014	0.513	0.158	0.834	0.005	0.786	0.012	0.696	0.037
Total water output (g/day/g BW)	−0.646	0.060	−0.357	0.346	0.028	0.943	0.103	0.792	0.421	0.260	0.168	0.666
Water intake (intake/g per g BW)	−0.621	0.074	−0.462	0.210	0.080	0.837	0.207	0.593	0.294	0.442	0.312	0.414
Food intake (intake/g per g BW)	0.657	0.055	0.593	0.093	−0.658	0.054	−0.488	0.183	−0.816	0.007	−0.742	0.022
Urine output (output/g per g BW)	−0.202	0.602	0.054	0.891	−0.268	0.486	−0.371	0.325	0.012	0.976	−0.218	0.572
Fractional urine output	0.311	0.415	0.524	0.147	−0.426	0.253	−0.708	0.033	−0.303	0.428	−0.611	0.081
Feces (output/g per g BW)	−0.711	0.032	−0.519	0.152	0.110	0.777	0.615	0.078	0.607	0.083	0.233	0.546
Urine osmolar excretion	0.752	0.019	0.772	0.015	−0.676	0.045	−0.801	0.009	−0.678	0.045	−0.898	0.001

*BW, bodyweight*.

## Discussion

Perfusion of human colon with bile acids results in secretion of water and electrolytes (18), and BAM can cause severe diarrhea. In the present study, we hypothesized that altered expression of AQPs in the colonic epithelium may be involved in the increased water fluxes elicited by bile acids. Our results demonstrate that variety of different AQPs, and several target proteins for bile acids, are expressed in colonic epithelial cells. Furthermore, we observed that bile acid administration to rats altered the expression of three of these AQPs (AQP3, AQP7, and AQP8), suggesting that these AQPs may be involved in the pathophysiology of bile acid-induced diarrhea.

The colon is highly water permeable, absorbing approximately 1.5–2 l of water each day *via* the crypt and surface epithelia (57). Various mechanisms for how water reabsorption occurs exist. Although paracellular transport along the osmotic gradient generated *via* the active transport of sodium by the Na-K-ATPase is thought to be a major route, tight epithelial layers of the colon suggest transcellular routes for water also exist. In this study, the identification of several AQPs in mouse, rat, and human colonic epithelial cells makes transcellular water transport *via* AQP water channels a distinct possibility. In particular, the clear segmental heterogeneity of predominant AQP3 expression in the distal colon could suggest a major role for this water channel in the dehydration of feces occuring in this segment. Furthermore, although others have identified AQPs in colons isolated from various species (45, 54, 58–69), our observations that AQP1 and AQP8 are localized to the apical plasma membrane and AQP3 or AQP4 are localized basolaterally in surface epithelial cells of the distal colon suggest that, in the segment where the majority of water reabsorption occurs (57, 70), a direct water transport pathway across epithelial cells exists. Such a role for AQPs in transepithelial water transport in the colon are supported by studies from AQP4 knockout mice, which have a significantly higher stool water content (71), and a study in rats where 1 h after rectally administered HgCL_2_ (non-selective AQP inhibitor) fecal water content was significantly increased (72).

Following bile acid treatment, AQP3, AQP7, and AQP8 were increased in mRNA expression alongside a concatenate increase in AQP7 and AQP8 protein levels. However, AQP3 protein levels decreased. This contradictory effect of bile acids on AQP3 mRNA expression and protein abundance may be a result of various posttranscriptional processes or protein degradation (73), or simply due to a biphasic response of AQP3 to bile acid exposure (initial downregulation of AQP3 followed by a compensatory upregulation). Roles for AQP3 and AQP8 in the pathogenesis of bile acid-induced diarrhea are supported by previous studies, with AQP3 mRNA levels increased in a cell model of secretory diarrhea induced by vasoactive intestinal polypeptide (released by enteric neurons) (74), and inhibition of AQP8 by siRNA in isolated superficial colonocytes resulting in decreased water permeability (65). Despite technical difficulties in localizing AQP7 in this study, its role in bile acid-induced diarrhea is also supported by previous studies localizing AQP7 to the apical membrane of surface cells within the colon (64, 75).

Previous studies of colonic AQP expression in various conditions of diarrhea support that AQPs may be differentially regulated by bile acids. For example, although we failed to detect mRNA for AQP3 in mice, others have reported that AQP3 and AQP8 mRNA levels increased in colonic scrapings isolated from the residual colon of mice subjected to resection of 80% of the distal small bowel (29). Such resection is likely accompanied by BAM. Interestingly, in conditions that cause diarrhea and are accompanied by inflammation, opposing effects on AQP expression have been observed. For example, it has been observed that (1) in a mouse model of rotavirus-induced diarrhea, the levels of AQP1, AQP4, and AQP8 are decreased, whereas AQP3 levels are increased (30); (2) in a mouse model of diarrhea induced by the chemotherapeutic drug 5-fluoracil and accompanied by an abnormal inflammatory response, the mRNA levels of AQP4 and AQP8 are decreased (76); (3) in humans or mouse models, ulcerative Crohn’s colitis or infectious colitis (inflammatory diseases) is accompanied by significant reductions in AQP4, AQP7, and AQP8 levels (64); (4) treatment of rats with rheinanthrone, which triggers macrophage activation, resulted in diarrhea and decreased expression of AQP3 (77); and (5) rectal treatment of rats with trinitrobenzene sulfonic acid to induce colitis (mimicking Crohn’s disease) resulted in reduced mRNA expression of AQP3 and AQP8 (78). Combined, these studies indicate that fundamentally different mechanisms, linked to the underlying cause of the diarrhea, may be responsible for the variable changes in AQP expression.

In theory, bile acids could be modulating the expression levels of AQPs in the colon by multiple pathways. Proteomic analysis of isolated colonic epithelial cells identified the FXR and VDR, which are known bile acid modulated steroid receptors. Therefore, it is possible that bile acids affect AQP gene transcription directly *via* these receptors. Bile acids may also indirectly affect AQPs in colonic epithelia *via* modulation of the enteric nervous system (ENS), perhaps *via* the enteroendocrine cells (79, 80) or *via* mast cell activation (81). Evidence for bile acids to acutely (within hours) promote secretion *via* the ENS has been documented in the small intestine of cat (82) and rat (83). As inflammation can be both a cause and an effect of diarrhea, and inflammation *per se* can affect the expression of AQPs, e.g., mimicking inflammatory bowel disease by treatment of intestinal cell cultures with IFNγ suppresses the expression of AQP1 (84), we cannot rule out that the changes in AQPs observed in our studies are due to secondary effects. Studies directly assessing the modulation of AQPs in cultured cells following stimulation with bile acids would help resolve this issue. Furthermore, it is also possible that alterations in AQP abundances occur *via* other indirect mechanisms, such as from bile acid-induced alterations in the colon microbiome. The composition of the microbiome is affected by the size of the bile acid pool and the composition of bile acids (85, 86), and although alterations in the microbiome may take a longer time than the period studied here, a previous study of germ-free mice and conventional mice indicated that AQP4 expression can be modulated *via* alterations in gut bacteria (87).

In summary, our studies demonstrate that AQPs have a heterogeneous expression pattern in colonic epithelial cells. During bile acid-induced diarrhea, the expression levels of AQP3, AQP7, and AQP8 are altered, suggesting that these channels are involved in the pathophysiology of BAM.

## Author Contributions

RF, SK, and HM conceived the initial idea behind the paper. JY, QW, SK, JB, NL, AO, RF, and HM performed scientific work, data analysis, and interpretation of data. PD provided access to and collected human for analysis. JY, RF, and HM drafted and edited the manuscript. JY, QW, SK, JB, NL, AO, PD, RF, and HM critically read the paper for final approval.

## Conflict of Interest Statement

The authors declare that the research was conducted in the absence of any commercial or financial relationships that could be construed as a potential conflict of interest.

## Footnotes

^1^https://hpcwebapps.cit.nih.gov/ESBL/ABE/

^2^http://interpretdb.au.dk/database/Colon/Mouse_Distal.html

^3^http://interpretdb.au.dk/database/Colon/Mouse_Proximal.html

^4^http://interpretdb.au.dk/database/Colon/Rat_Distal.html

^5^http://interpretdb.au.dk/database/Colon/Rat_Proximal.html

## Supplementary Material

Data Sheet S1 **Mouse and rat colon proteome**.

Data Sheet S2 **Mouse GO-term analysis**.

Data Sheet S3 **Rat GO-term analysis**.
